# Results of transarterial chemoembolization of hepatocellular carcinoma as a bridging therapy to liver transplantation

**DOI:** 10.1590/0100-3984.2023.0040

**Published:** 2023

**Authors:** Raquel de Freitas Jotz, Alex Finger Horbe, Gabriela Perdomo Coral, Priscila Cavedon Fontana, Beatriz Garcia de Morais, Angelo Alves de Mattos

**Affiliations:** 1 Universidade Federal de Ciências da Saúde de Porto Alegre (UFCSPA), Porto Alegre, RS, Brazil; 2 Irmandade Santa Casa de Misericórdia de Porto Alegre (ISCMPA), Porto Alegre, RS, Brazil

**Keywords:** Chemoembolization, therapeutic, Necrosis, Carcinoma, hepatocellular, Survival analysis., Quimioembolização terapêutica, Necrose, Carcinoma hepatocelular, Análise de sobrevida.

## Abstract

**Objective:**

To evaluate the degree of tumor necrosis after transarterial chemoembolization (TACE), used as a bridging therapy in patients awaiting liver transplantation, and its effect on survival.

**Materials and Methods:**

This was a retrospective cohort study involving 118 patients submitted to TACE prior to liver transplantation, after which the degree of tumor necrosis in the explant and post-transplant survival were evaluated.

**Results:**

Total necrosis of the neoplastic nodule in the explant was observed in 76 patients (64.4%). Of the patients with total necrosis in the explanted liver, 77.8% had presented a complete response on imaging examinations. Drug-eluting bead TACE (DEB-TACE), despite showing a lower rate of complications than conventional TACE, provided a lower degree of total necrosis, although there was no statistical difference between the two. By the end of the study period, 26 of the patients had died. Survival was longer among the patients with total necrosis than among those with partial or no necrosis (HR = 2.24 [95% CI: 0.91-5.53]; *p* = 0.078).

**Conclusion:**

In patients undergoing TACE as a bridging therapy, total tumor necrosis appears to be associated with improved patient survival.

## Abstract (pt)

**Objetivo:**

Avaliar os resultados da necrose tumoral após quimioembolização transarterial (TACE) como terapia ponte e seu reflexo na sobrevida dos pacientes.

**Materiais e Métodos:**

Estudo de coorte retrospectivo, com 118 pacientes que realizaram TACE, em que foram avaliados o grau de necrose tumoral no explante e a sobrevida pós-transplante.

**Resultados:**

Necrose total do nódulo neoplásico no explante foi observada em 76 pacientes (64,4%). Observou-se que 77,8% dos pacientes com necrose total no explante hepático tinham apresentado resposta completa nos exames de imagem. A DEB-TACE, apesar de ter demonstrado menor taxa de intercorrências, proporcionou menor grau de necrose total em relação à TACE convencional, a despeito de não haver diferença estatística. Ao final do seguimento do estudo, o número de óbitos foi de 26. A sobrevida foi maior nos pacientes que tiveram necrose total quando comparada com grau de necrose parcial ou ausência de necrose [HR = 2,24 (IC 95%: 0,91-5,53); *p* = 0,078].

**Conclusão:**

Necrose completa do tumor nos pacientes submetidos a TACE como terapia ponte parece estar associada com melhora da sobrevida.

## INTRODUCTION

Hepatocellular carcinoma (HCC) accounts for approximately 75-85% of cases of primary liver neoplasms^**(^1^)**^. Despite the various existing therapeutic proposals, liver transplantation is the ideal treatment because it removes the cancer and provides a cure for the underlying chronic liver disease. The significant limitation in its indication is the disproportionality between the number of donors and that of patients awaiting transplantation. Therefore, it should be indicated judiciously^**(^2^-^4^)**^.

Within the medical community, there is great concern regarding the possibility of patient dropout from transplant waiting lists. For patients on the waiting list for liver transplantation, failure to provide a bridging therapy has been shown to result in a dropout rate as high as 25% in six months and 38% in one year^**(^5^)**^. When the time on the waiting list exceeds six months, locoregional therapy is recommended^**(^5^-^7^)**^. The aim of such therapy is to control the tumor, improve transplant outcomes, and exclude HCCs that are biologically more aggressive^**(^8^)**^. However, there is controversy regarding this practice^**(^8^-^11^)**^.

Locoregional therapies, such as transarterial embolization, transarterial chemoembolization (TACE), transarterial radioembolization, local ablation therapies, stereotactic body radiation, and combinations of those strategies, have been considered for use as bridging therapies. As a rule, those pre-transplant therapies do not provoke severe adverse events, although minor complications reportedly occur in 2.3-8.3% of cases^**(^12^,^13^)**^. No superiority has been demonstrated among the proposed methods^**(^5^)**^. However, among the procedures used as bridging therapies, we highlight TACE^**(^14^)**^ because it is the most widely used. There are two types of TACE techniques-conventional TACE and drug-eluting bead TACE (DEB-TACE)-and the latter is theoretically associated with fewer toxic systemic effects^**(^15^)**^. Most studies have shown no significant difference between the two techniques in terms of patient survival rates^**(^15^,^16^)**^.

The objective of the present study is to assess the degree of tumor necrosis after TACE, used as a bridging therapy, in patients on the waiting list for liver transplantation. A secondary objective was to evaluate the effect that the use of TACE has on post-transplant patient survival.

## MATERIALS AND METHODS

This was a retrospective cohort study that included patients ≥ 18 years of age with a diagnosis of HCC who underwent TACE, as a bridging therapy to liver transplantation, between January 2013 and December 2021 at a public tertiary hospital in southern Brazil. The study was approved by the Research Ethics Committee of the Irmandade Santa Casa de Misericórdia de Porto Alegre, in the city of Porto Alegre, Brazil.

The diagnosis of HCC was established by using triple-phase computed tomography or gadolinium contrast-enhanced magnetic resonance imaging as dynamic imaging techniques, in accordance with the guidelines established by the American Association for the Study of Liver Diseases^**(^5^)**^. When necessary, a liver biopsy was performed.

When the expected time on the waiting list was more than six months, TACE was recommended. In all cases, conventional TACE or DEB-TACE was carried out by an experienced interventional radiologist. Superselective catheterization was performed in most cases. The chemotherapy drug utilized was doxorubicin (1 mL diluted in 2-3 mL of lipiodol). Patients undergoing TACE as a definitive palliative therapy or for an indication other than the treatment of HCC were excluded, as were those for whom the data in the medical record were incomplete.

Of 480 eligible patients, 136 met the inclusion criteria. The histology report was missing in 18 cases. Therefore, the final sample comprised 118 patients.

The following patient characteristics were evaluated: age, gender, etiology of cirrhosis, and Child-Pugh score. Patients were classified, regarding whether or not liver transplantation was indicated, according to the Milan criteria^**(^3^)**^.

For HCCs, the variables studied were the number of nodules, the diameter of the largest nodule, the presence of portal vein thrombosis, and the alpha-fetoprotein (AFP) level at the time of diagnosis. An AFP cut-off of 100 ng/mL has previously been established^**(^17^)**^.

The arterial chemoembolization procedure was evaluated regarding the number of sessions, chemotherapeutic drugs used, complications after TACE, and response to TACE. The response to TACE was evaluated as described in the Modified Response Evaluation Criteria in Solid Tumor (mRECIST) guidelines^**(^18^)**^ and was correlated with the degree of tumor necrosis of HCC in the liver explant.

In the explanted liver, the degree of tumor differentiation (when total necrosis of the lesion was not achieved), the presence of satellite nodules, and microvascular invasion, as well as the degree of necrosis observed in the largest lesion, were evaluated by an experienced liver pathologist. In assessing the degree of tumor differentiation, we used the histological classification devised by the Liver Cancer Study Group of Japan^**(^19^)**^.

The number of deaths, in relation to the degree of necrosis of the liver explant, was recorded. The patients were followed until death or until the last outpatient visit in December 2021. Survival was calculated from the time of liver transplantation.

Quantitative data are expressed as mean, standard deviation, and range. Categorical data are expressed as absolute values and percentages. Quantitative data were compared between groups by using analysis of variance or the Kruskal-Wallis test. For categorical data, we used Fisher’s exact test for comparisons. Poisson regression was used to compare event counts and to estimate incidence densities. Kaplan-Meier curves and a Cox regression model were used for the survival analysis. Values of *p* < 0.05 were deemed statistically significant. All analyses were conducted with the IBM SPSS Statistics software package, version 25.0 (IBM Corp., Armonk, NY, USA).

## RESULTS

Initially, 136 patients were included in the study. The sociodemographic and clinical characteristics of the patients can be seen in Table 1. The degree of necrosis was evaluated by consulting the description of the explanted liver on the histopathology reports. Among the 118 patients selected for analysis, total necrosis in the explant was reported in 76 (64.4%). The degree of liver necrosis was not found to correlate with the etiology of liver disease (Table 2).

**Table 1:** Sociodemographic and clinical characteristics of patients undergoing chemoembolization and liver transplantation.

Characteristic	(n = 136)
Age (years), mean ± SD	61.5 ± 7.0
Gender, n (%)	
Male	103 (75.7)
Female	33 (24.3)
Cirrhosis etiology, n (%)	
Hepatitis C virus	77 (56.6)
Hepatitis C virus+alcoholic liver disease	24 (17.6)
Alcoholic liver disease	11 (8.1)
Nonalcoholic steatohepatitis	10 (7.4)
Hepatitis B virus	6 (4.4)
Hepatitis C virus+hepatitis B virus	3 (2.2)
Hepatitis B virus+alcoholic liver disease, hemochromatosis,	
or cryptogenesis	5 (3.7)
Child-Pugh score, n (%)^*^	
A	104 (79.4)
B+C	27 (20.6)

* Data available for only 131 patients. SD, standard deviation.

**Table 2:** Characteristics of patients, by the degree of necrosis observed in the histopathological examination of the liver explant (n = 118).

Characteristic	Degree of necrosis			*P*
	None (n = 13)	Partial (n = 29)	Total (n = 76)	
Age (years), mean ± SD	59.5 ± 6.9	62.4 ± 6.9	62.0 ± 7.0	0.428^*^
Male, n (%)	9 (69.2)	25 (86.2)	57 (75.0)	0.343^†^
Cirrhosis etiology, n (%)				0.852^†^
Hepatitis C virus	8 (61.5)	15 (51.7)	43 (56.6)	
Hepatitis C virus+alcoholic liver disease	2 (15.4)	6 (20.7)	14 (18.4)	
Alcoholic liver disease	1 (7.7)	4 (13.8)	5 (6.6)	
Nonalcoholic steatohepatitis	2 (15.4)	2 (6.9)	4 (5.3)	
Hepatitis B virus	-	-	5 (6.6)	
Hepatitis C virus+hepatitis B virus	-	-	2 (2.6)	
Hepatitis B virus+alcoholic liver disease, hemochromatosis, or cryptogenesis	-	2 (6.9)	3 (3.9)	
Child-Pugh score A, n/total (%)	10/13 (76.9)	21/28 (75.0)	60/73 (82.2)	0.685^†^
Milan criteria, n/total (%)				0.024^†^
1 nodule ≤ 5 cm	5/10 (50.0)	8/26 (30.8)	42/68 (61.8)	
2-3 nodules ≤ 3 cm	5/10 (50.0)	18/26 (69.2)	26/68 (38.2)	
Number of nodules. mean ± SD	3.54 ± 3.18	2.69 ± 2.35	2.00 ± 1.56	0.002^‡^
Size of the largest nodule (cm), mean ± SD	2.73 ± 1.50	3.08 ± 0.95	2.96 ± 1.20	0.540^§^
AFP ≥ 100 ng/mL, n/total (%)	3/11 (27.3)	4/23 (17.4)	13/63 (20.6)	0.862^†^
Portal thrombosis, n/total (%)	0/13 (0.0)	3/29 (10.3)	6/74 (8.1)	0.587^†^
TACE number	1.1	1.3	1	< 0.001^†^
Degree of tumor differentiation, n/total (%)				> 0.99
1	1/10 (10.0)	4/27 (14.8)	-	
2	8/10 (80.0)	21/27 (77.8)	-	
3	1/10 (10.0)	2/27 (7.4)	-	
Microvascular invasion, n/total (%)	2/13 (15.4)	8/29 (27.6)	4/75 (5.3)	0.004^†^
Satellite nodules, n/total (%)	5/13 (38.5)	5/29 (17.2)	12/74 (16.2)	0.216^†^
mRECIST response, n/total (%)				< 0.001
Complete	2/12 (16.7)	12/26 (46.2)	56/72 (77.8)	
Partial	5/12 (41.7)	12/26 (46.2)	13/72 (18.1)	
Stable or progressive disease	5/12 (41.7)	2/26 (7.7)	3/72 (4.2)	
Death, n (%)	1 (7.7)	9 (31.0)	9 (11.8)	0.048^†^

* Analysis of variance.

† Fisher’s exact test.

‡ Poisson regression.

§ Kruskal-Wallis test. SD, standard deviation.

Applying the Milan criteria, we identified a statistically significant difference, in terms of the degree of necrosis, between the patients with only one nodule ≤ 5 cm and those with two or three nodules ≤ 3 cm (*p* = 0.024); the proportion of patients with total necrosis in the explant after chemoembolization was greater among those with only one nodule ≤ 5 cm (61.8% vs. 38.2%). This was also confirmed in the analysis of the number of nodules in relation to the degree of necrosis after TACE, a lower number of nodules prior to chemoembolization having been found to be statistically significant for achieving total necrosis in the explant (*p* = 0.002). There was no statistically significant difference regarding the size of the nodule after chemoembolization.

In the AFP analysis, when the cut-off of 100 ng/mL was used, there was no significant difference in the degree of necrosis. The mean number of chemoembolization procedures performed in patients with no, partial, and total necrosis was 1.1, 1.3, and 1, respectively. There was no statistical difference between the patients with no necrosis (n = 10) and those with partial necrosis (n = 27), in terms of the degree of tumor differentiation (*p* > 0.99).

Most of the patients with total necrosis after undergoing the procedure had no microvascular invasion in the explant (*p* = 0.004). There was no correlation between total necrosis and a lower number of satellite nodules.

**Figure 1:**
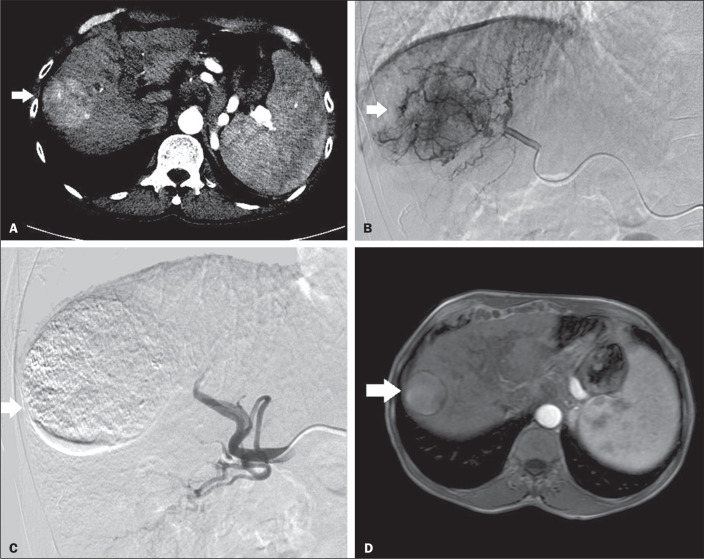
A: Computed tomography scan showing a LI-RADS category 5 lesion in the right lobe. B: Superselective angiography showing a hypervascular lesion. C: Follow-up angiography, performed at the end of the procedure, showing the devascularized lesion. D: Follow-up magnetic resonance imaging scan, acquired 60 days after the procedure, showing no gadolinium uptake by the tumor.

When we looked for a correlation between the mRECIST response and total necrosis in the explant, we found that 77.8% of the patients with total necrosis in the explanted liver had presented a complete response by the mRECIST classification (*p* < 0.001). Computed tomography was the method utilized for analyses of the mRECIST response in most patients (in some cases magnetic resonance imaging was utilized), which was performed between one and two months after TACE. The time between imaging and liver transplantation was not more than five months. Figure 1 shows a complete response of the tumor after TACE.

The particles used in chemoembolization therapy were also analyzed and compared regarding their effectiveness in promoting necrosis in the tumor (Table 3). Polyvinyl alcohol, Embosphere, and Bead Block microspheres produced better results than did the HepaSphere microspheres (used in DEB-TACE). The HepaSphere particle provided total necrosis in 43.8% of the cases, compared with approximately 70% for the other particles, although there was no statistical difference. The rate of complications was lower for use of the HepaSphere particle, but, again, there was no statistical difference (*p* = 0.465).

**Table 3:** Comparison of particles used in the chemoembolization procedure for HCCs.

Variable	HepaSphere^*^	Embosphere	Polyvinyl alcohol	Bead Block	*P*
Degree of necrosis, n/total (%)					0.203
Total	14/32 (43.8)	27/39 (69.2)	19/25 (76.0)	13/17 (76.5)	
Partial	12/32 (37.5)	8/39 (20.5)	4/25 (16.0)	3/17 (17.6)	
None	6/32 (18.8)	4/39 (10.3)	2/25 (8.0)	1/17 (5.9)	
Complication(s), n/total (%)	3/39 (7.7)	4/45 (8.9)	3/26 (11.5)	4/19 (21.1)	0.465
Child-Pugh score, n/total (%)					0.432
A	29/38 (76.3)	35/43 (81.4)	21/26 (80.8)	12/17 (70.6)	
B	9/38 (23.7)	8/43 (18.6)	5/26 (19.2)	5/17 (29.4)	
Milan criteria, n/total (%)					0.762
1 nodule ≤ 5 cm	17/32 (53.1)	23/42 (54.8)	10/23 (43.5)	11/18 (61.1)	
2-3 nodules ≤ 3 cm	15/32 (46.9)	19/42 (45.2)	13/23 (56.5)	7/18 (38.9)	

* DEB-TACE.

Of the 136 patients evaluated, 16 (11.76%) had complications related to TACE: abdominal pain was the most common. Two of those patients had post-chemoembolization syndrome, but they recovery satisfactorily.

**Figure 2:**
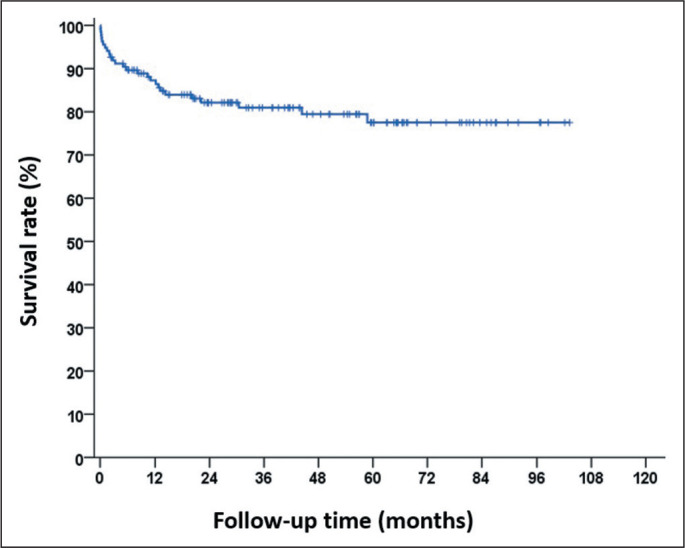
Survival rate and follow-up time.

When we analyzed the sample as a whole, using the Kaplan-Meier curve, we observed that the survival rate was 87.3% at one year, 82.1% at two years, 80.9% at three years, and 77.5% at five years. The median follow-up time was 43.7 months (Figure 2).

**Figure 3:**
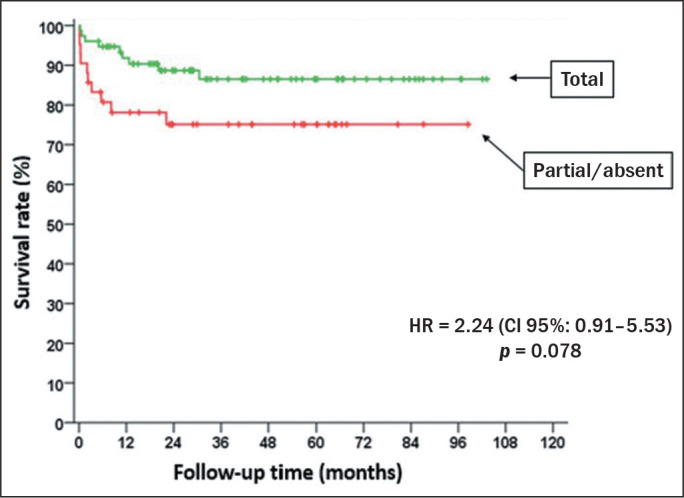
Survival rate in relation to the degree of necrosis and follow-up time.

By the end of the study follow-up period, 26 of the patients had died. The leading causes of death were postoperative complications of liver transplantation, in 12 (46.2%), causes unrelated to the tumor (infection), in 10 (38.5%), and progressive neoplastic disease, in 4 (15.4%). Because the number of deaths among the patients without necrosis was very small, they were grouped with those among the patients with partial necrosis, and there was a trend toward lower mortality among the patients with total necrosis than among those with partial/no necrosis. Of the 42 patients without total necrosis in the explant, 10 (23.8%) died, compared with only nine (11.8%) of the 76 with total necrosis. Therefore, as shown in Figure 3, the mortality rate in the group of patients with total necrosis (2.8 deaths/1,000 patient-months) was lower than was that in the group without (6.97 deaths/1,000 patient-months), with a hazard ratio of 2.24 (95% CI: 0.91-5.53), although the difference was less than significant (*p* = 0.078).

## DISCUSSION

HCC is the sixth most common malignant neoplasm and the third leading cause of cancer-related death worldwide. It is a significant cause of morbidity and mortality in patients with cirrhosis^**(^20^)**^.

Liver transplantation is the ideal treatment for patients with HCC. However, the shortage of organs available for donation requires careful prioritization of patients on the transplant waiting list. Locoregional therapy has been offered to waitlisted patients to avoid dropout^**(^5^-^7^,^13^)**^. Nevertheless, there is no consensus regarding this approach in the literature, mainly because of the difficulty of performing randomized, controlled prospective studies^**(^5^,^12^,^21^,^22^)**^.

In the present study, in which we analyzed a sample of more than 100 patients undergoing TACE as a bridging therapy, total tumor necrosis in the explant did not differ significantly from incomplete necrosis in terms of survival, despite the longer survival of the patients with total necrosis. As in other studies in the Western literature, the average age of the patients was approximately 60 years, most of the patients were male, and the most common etiology of cirrhosis was infection with hepatitis C virus. The most representative Child-Pugh score was A, and most patients also met the Milan criteria^**(^23^-^25^)**^.

In our study sample, total necrosis was observed mainly in the patients with a solitary nodule. In the patients with multiple nodules, achieving total necrosis was related to a lower number of nodules. Other authors have also reported that the achievement of total necrosis is more common among patients with a solitary nodule^**(^26^)**^. In the present study, there was no difference in the degree of necrosis in relation to the size of the largest nodule, the degree of tumor differentiation (although this was evaluated only in the patients without total necrosis), or the AFP level. Some authors consider a low AFP level to be an independent predictor of total necrosis^**(^26^,^27^)**^. Of the patients with total necrosis in our sample, most had no microvascular invasion in the explant, suggesting that the risk of vascular invasion was lower in that group of patients. The mean number of procedures performed in our sample was lower than the 2.5 ± 1.5 reported in the literature^**(^28^)**^.

The reported level of interobserver agreement between radiologists for the presence or absence of a Liver Imaging Reporting and Data System (LI-RADS) category 5 lesion was excellent when mRECIST criteria were utilized^**(^29^)**^. In the evaluation of the tumor response after TACE, according to the mRECIST classification, a correlation has been observed between the mRECIST response and total necrosis in the liver explant, nearly 80% of patients with total necrosis in the explant having been found to show a complete response according to the mRECIST classification^**(^19^)**^.

In the present study, survival was greater among the patients with total necrosis, although the difference in comparison with the other patients did not reach statistical significance, which is probably due to the relatively small sample size. The mortality rate was lower among the patients with total necrosis than among those without. Similar findings were reported by Allard et al.^**(^26^)**^, although their study demonstrated a lower incidence of tumor recurrence in patients with total necrosis. A multicenter study analyzing a large patient sample showed that when total necrosis was achieved, survival was longer and the tumor recurrence rate was lower^**(^8^)**^. However, when the sample as a whole was evaluated, no difference in the outcomes was observed between the patients who underwent bridging therapy and those who did not, regardless of the degree of necrosis achieved. Similar results regarding total necrosis were obtained in another large multicenter study^**(^28^)**^. It should be borne in mind that those studies also evaluated other modalities of locoregional therapy and not only TACE.

Systematic reviews focusing on bridging therapy are generally of poor quality, showing either improved survival when bridging therapy is used^**(^22^)**^ or demonstrating its ineffectiveness^**(^23^)**^. Recently, Butcher et al.^**(^30^)**^ showed that individuals treated with TACE, despite having worse prognostic characteristics (in terms of tumor diameter and longer time on the waiting list), had survival and postoperative outcomes similar to those of patients who did not undergo bridging therapy. However, their analysis included patients with tumors that had been downstaged. The most recent systematic review and meta-analysis on the topic in question^**(^12^)**^, using the concept of intention-to-treat in an original way, concluded that patients undergoing bridging therapy before liver transplantation, despite being on the waiting list for longer than those who did not undergo the procedure, had better post-transplant survival. Nevertheless, when the intention-to-treat analysis was performed, there was no difference between those two groups in terms of the one-, three-, or five-year survival rate.

As limitations in the present study, we call attention mainly to its retrospective nature, the fact that we did not evaluate the incidence of tumor recurrence, and the relatively small number of patients evaluated. If we had analyzed a larger cohort, the better survival of patients with total necrosis in the explant might have reached statistical significance.

## CONCLUSION

This real-life study revealed that, in patients undergoing TACE as a bridging therapy, total tumor necrosis appears to be associated with improved patient survival. However, prospective controlled studies are needed in order to obtain a more definitive answer regarding the best practice in patients on the waiting list for liver transplantation.
